# The Vapor Controversy

**Published:** 1847-07

**Authors:** 


### EDITORIAL DEPARTMENT.

*The Vapor Controversy.*—The contest for the credit of the discovery of the pain-preventing vapor, like the Mexican war, seems less likely to terminate the more it progresses. We referred, in our last number, to a pamphlet, published by Mr. Horace Wells, of Hartford, with the view of substantiating his claim to priority; within a few days, we have received another, by Dr. Gay, of Boston, in advocacy of the claims of Dr. Jackson. From a survey of all the circumstances pertaining to the history of the rise and progress of the discovery, in so far as they have come to our knowledge, it appears to us that the credit does not belong exclusively to either of the claimants, but, measurably, to all. Mr. Wells, it would seem, was the first to be impressed with the conviction that *inhalations* might be rendered available for the object, sufficiently to institute a series of experiments, and to endeavor to enlist the interest of the profession in the subject. His experiments were, for the most part, made with the nitrous oxide; yet, in so far as the merit of orginality in the *idea* is concerned, it may be fairly questioned if the choice of exhilarating vapor is a very important circumstance. Moreover, Mr. Wells adduces evidence to show that in one instance, at least, he made trial of the ether, but was led to abandon it. Mr. Wells' efforts, however, failed in exciting much attention. In his publication, he would imply that Drs. Jackson and Morton derived the idea from him. With reference to the former of these gentlemen, we may be permitted to say, that few, if any, who know the position which he occupies, as a man of science, and a gentleman, will be ready to believe that he would enact the part of a plagiarist in this, or any other matter. We do not doubt that with Dr. Jackson the idea was original, and that he is entitled to the merit of originality; but he does not appear to have appreciated, fully, its importance, else it is inconceivable that he should have been so dilatory in instituting observations, and in bringing his discovery before the profession. The information which, as represented in Dr. Gay's pamphlet, Dr. Jackson communicated to several individuals, seems to have been given in a casual manner, very unlike, as one would suppose, the enunciation of a fact so novel in its character, and so important to humanity, as we presume Dr. J., as well as others, now regards the discovery to be.

Dr. Morton appears to have a participation in the matter rather practical than inventive. It is claimed for him that he was indebted to no one for the suggestion of the ether; but we do not recollect that any testimony is adduced in support of this statement, and the evidence presented by Dr. Gay goes to show that he was unacquainted with the properties of ether prior to the hint furnished by Dr. Jackson. We must say this fact involves a degree of ignorance hardly imaginable in one who prefixes the title of *Dr.* to his name, and we were somewhat inclined to suspect that it was feigned, in order to subserve some particular ends. At all events, Dr. Morton was not slow in perceiving that the idea was worth acting upon, and that without delay; and the credit of carrying it into practice, demonstrating its success, and bringing it into adoption, seems due to him.

In the present posture of affairs, something like a *pro rata* division of the discovery appears to us most reasonable, and just to all parties; but, if either of the disputants are really entitled to it exclusively, we sincerely hope that in the end it will so appear, and that the profession will be able to give the honor to him to whom it appropriately belongs.

